# Relative abundance of *Akkermansia* spp. and other bacterial phylotypes correlates with anxiety- and depressive-like behavior following social defeat in mice

**DOI:** 10.1038/s41598-019-40140-5

**Published:** 2019-03-01

**Authors:** Kara D. McGaughey, Tulay Yilmaz-Swenson, Nourhan M. Elsayed, Dianne A. Cruz, Ramona M. Rodriguiz, Michael D. Kritzer, Angel V. Peterchev, Jeffrey Roach, William C. Wetsel, Douglas E. Williamson

**Affiliations:** 1 Department of Psychiatry and Behavioral Sciences, Duke University Medical Center, Durham, NC 27710 USA; 2 Mouse Behavioral and Neuroendocrine Analysis Core Facility, Duke University Medical Center, Durham, NC 27710 USA; 3 Department of Biomedical Engineering, Duke University, Durham, NC 27708 USA; 4 Department of Electrical and Computer Engineering, Duke University, Durham, NC 27708 USA; 5 Department of Neurosurgery, Duke University School of Medicine, Durham, NC 27710 USA; 6 Research Computing, University of North Carolina at Chapel Hill, Chapel Hill, NC 27599 USA; 7 Durham VA Medical Center, Durham, NC 27705 USA

## Abstract

As discussion of stress and stress-related disorders rapidly extends beyond the brain, gut microbiota have emerged as a promising contributor to individual differences in the risk of illness, disease course, and treatment response. Here, we employed chronic mild social defeat stress and 16S rRNA gene metagenomic sequencing to investigate the role of microbial composition in mediating anxiety- and depressive-like behavior. In socially defeated animals, we found significant reductions in the overall diversity and relative abundances of numerous bacterial genera, including *Akkermansia* spp., that positively correlated with behavioral metrics of both anxiety and depression. Functional analyses predicted a reduced frequency of signaling molecule pathways, including G-protein-coupled receptors, in defeated animals. Collectively, our data suggest that shifts in microbial composition may play a role in the pathogenesis of anxiety and depression.

## Introduction

Major depressive disorder (MDD) is a debilitating and stigmatized public health concern. While recognized as a leading cause of disability worldwide and affecting up to 16% of the population, definitive mechanisms underlying the pathophysiology of MDD remain unknown^1–3^. Enabled by the emergence of molecular and metagenomic technologies, the focus of mental health has rapidly extended to include not only the central nervous system, but also its connection to and communication with the periphery, namely the gut microbiome. Unsurprisingly, given the high comorbidity between gastrointestinal disorders and depression, studies have revealed that patients with MDD have an altered microbial composition^4–6^. This finding has been recapitulated in various animal models as exposure to social stressors known to elicit anxiety- and depressive-like behavior have been shown to produce alterations in the structure of gut microbial communities^7–11^.

However, the microbiota-gut-brain axis is bidirectional. Not only are anxiety and depression reflected in microbial composition, but evidence suggests that microbiota can influence brain function and behavior^4,12–15^. Colonization of germ-free mice with ‘depressive’ fecal microbiota from patients with MDD has produced depressive-like behaviors compared to colonization with ‘healthy’ microbiota from control subjects^4^. As such, the composition of gut microbial communities becomes an increasingly recognized environmental factor contributing to individual differences in risk of illness, disease course, and treatment response.

This study investigated whether exposure to stress disrupted gut microbiota in a preclinical model of depression. We used 16S rRNA gene sequencing to examine shifts in bacterial communities resulting from a mild social defeat stress at the phylum and genus taxonomic ranks. Here, we show that shifts in the abundance of particular bacterial genera, like *Akkermansia*, *Ruminococcus*, and *Dorea*, resulted from exposure to this social stressor and correlated with anxiety- and depressive-like behavior.

## Results

### Exposure to mild chronic social defeat stress is sufficient to induce anxiety- and depressive-like behaviors

**Figure 1:**
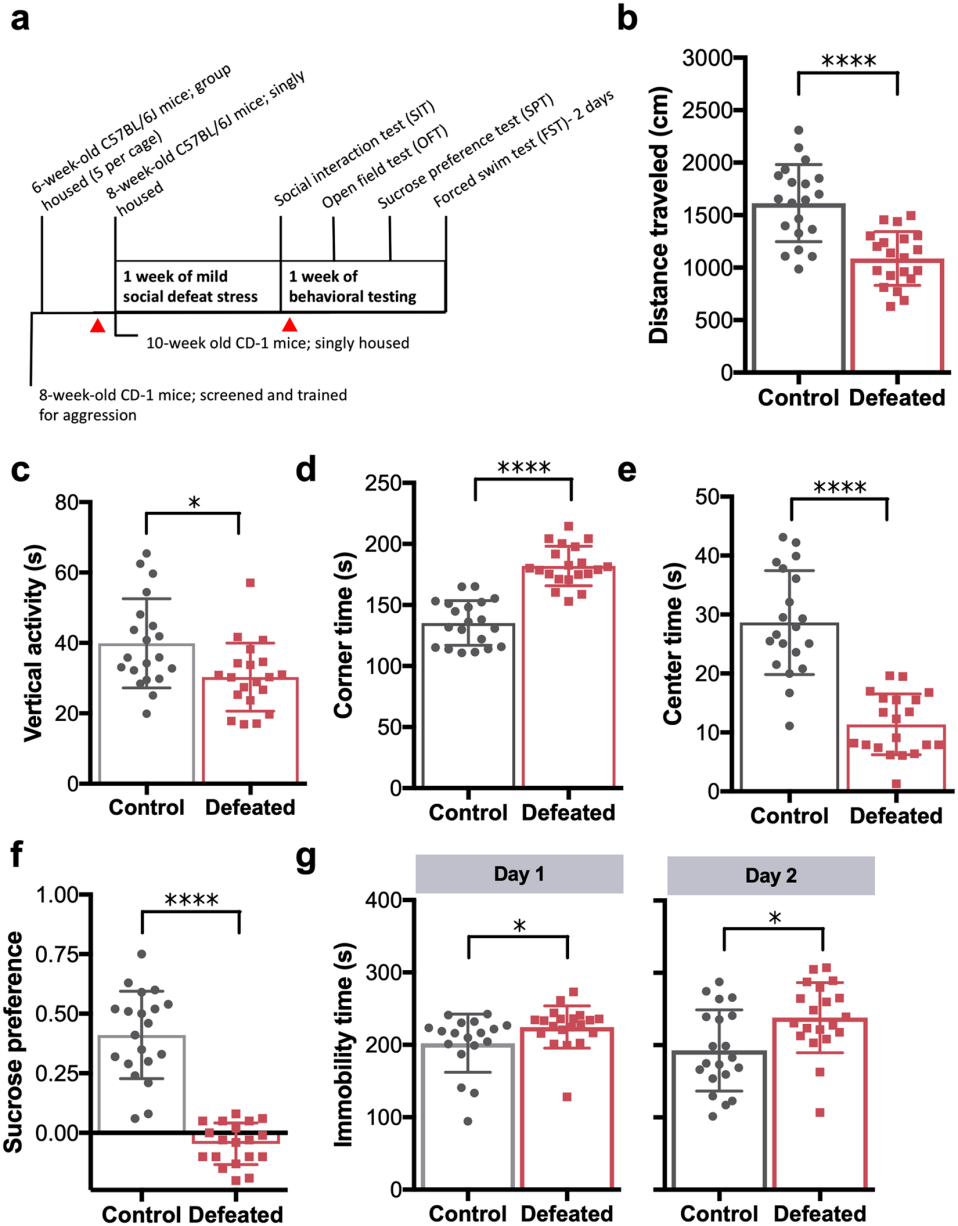
Chronic mild social defeat stress induces anxiety- and depressive-like behavior. (**a**) Experimental design. Red arrows indicate time points of fecal sample collection. (**b**–**e**) Quantification of behavior during the open field test [n = 20 control and 20 defeated; Wilcoxon rank sum test, *p < 0.05, ****p < 0.0001; mean ± standard deviation (s.d.)]. (**f**) Sucrose preference (n = 20 control and 19 defeated; Wilcoxon rank sum test, ****p < 0.0001; mean ± s.d.). (**g**) Immobility behavior during the two-day forced swim test (day 1: n = 18 control and 20 defeated, day 2: n = 20 control and 20 defeated; Wilcoxon rank sum test, *p < 0.05; mean ± s.d.).

To induce anxiety- and depressive-like behaviors, we employed a modified model of chronic social defeat stress. In an attempt to minimize physical wounding of the mice and subsequent, confounding effects of inflammation on microbiota, we adapted previous protocols^16^ to include fewer sessions of defeat. (Fig. 1a). Despite exposure to this milder iteration of chronic stress, defeated animals still displayed expected behavioral alterations. During the open field test, for example, defeated mice exhibited reductions in distance traveled (*p* < 0.0001) as well as vertical activity (*p* < 0.05) (Fig. 1b,c), consistent with lower levels of exploratory behavior. Milder social defeat also induced anxiety-like behavior, as demonstrated by decreased center time (*p* < 0.0001) and increased corner time (*p* < 0.0001) (Fig. 1d,e). Defeated animals also displayed significant increases in depressive-like behaviors, such as anhedonia and despair, as evidenced by decreased sucrose preference (*p* < 0.0001) as well as increased immobility time in the forced swim test (Day 1: *p* < 0.05; Day 2: *p* < 0.05) (Fig. 1f,g). Food and water intake among the control and defeated animals varied throughout the paradigm. However, body weights assessed pre- and post-social defeat exposure were comparable (see Supplementary Fig. S1).

### Microbiota composition is altered by chronic mild social defeat stress

**Figure 2:**
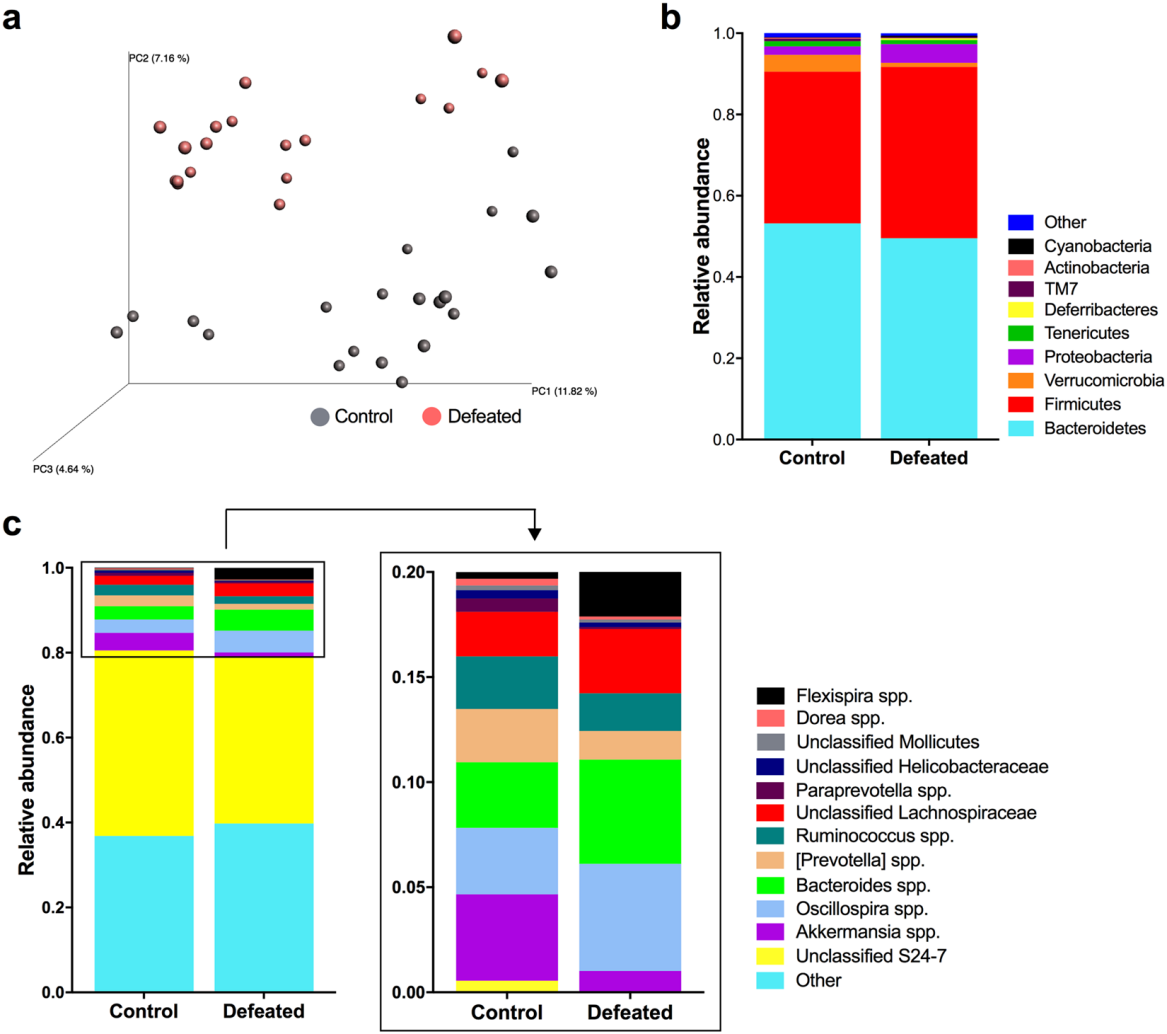
Exposure to chronic mild social defeat stress induces shifts in microbial communities and alters the abundance of relevant taxa. (**a**) Principal coordinate analysis of the bacterial communities among the control (gray) (n = 19) and defeated (red) (n = 20) mice. Fecal samples were collected 24 hr after the social interaction test, which immediately followed social defeat or the control condition. (**b**) Relative abundances of the bacterial communities at the phylum taxonomic rank among the control and defeated mice. (**c**) Representative graphs of the relative abundances of the bacterial communities at the genus taxonomic rank among the control and defeated mice. Defeated animals showed decreases in *Akkermansia* spp. (light purple), [*Prevotella*] spp. (tan), *Ruminococcus* spp. (dark green), *Paraprevotella* spp. (dark purple), unclassified *Helicobacteraceae* (dark blue), *Dorea* spp. (pink), and *Mollicutes* (gray) as well as increases in *Oscillospira* spp. (light blue), *Bacteroides* spp. (light green), unclassified *Lachnospiriaceae* (red), and *Flexispira* spp. (black).

To explore the relationship between stressor-induced changes in behavioral phenotype and the microbiome, we used fecal samples to profile the structural composition of the microbiota. At baseline, microbial composition did not differ between to-be control and to-be defeated animals (see Supplementary Fig. S2). Following mild exposure to social defeat stress, analysis of alpha-diversity revealed a pronounced decrease in the richness of the microbial community, resulting in lower estimates of species and operational taxonomic units (OTUs) (Chao1: *p* < 0.05; observed OTUs: *p* < 0.05; see Supplementary Fig. S3). However, the diversity of microbial composition in the defeated animals was comparable to that of the controls (Shannon: *p* = 0.291; see Supplementary Fig. S3). Clustering of gut microbiota by stress exposure group was observed with unweighted Unifrac principal coordinate analysis (PCoA) revealing shorter distances between intra-group samples than between group samples (999 permutations, non-parametric *p* < 0.01) (Fig. 2a). These results were confirmed using an analysis of similarities (ANOSIM) test on the unweighted Unifrac distances (10,000 permutations, *p* < 0.0001).

The relative abundances of the bacterial populations in the *Bacteroidetes* and *Firmicutes* phyla, which accounted for approximately 90% of the identified sequences, were comparable between the control and defeated animals (*p* = 0.189 and *p* = 0.061, respectively) (Fig. 2b). However, the mild chronic social defeat stress did alter the relative abundances of four of the nine phyla, resulting in decreases in *Actinobacteria* (control *vs*. defeated: 0.1% *vs*. 0.0%; *p* < 0.001) and *Verrucomicrobia* (control *vs*. defeated: 4.1% *vs*. 1.0%; *p* < 0.001) as well as increases in *Deferribacteres* (control *vs*. defeated: 0.0% *vs*. 0.2%; *p* < 0.001) and *Proteobacteria (*control *vs*. defeated: 2.1% *vs*. 4.6%; *p* < 0.005), which accounted for approximately 0.1%, 2.4%, 0.1%, and 3.3% of the identified sequences, respectively (Table 1).

**Table 1:** Relative abundances of bacterial phyla in control *vs*. mice exposed to chronic mild social defeat stress.

Phylum	Control (n = 19)	Defeated (n = 20)	*p value*
Deferribacteres	0 ± 0	0.0020 ± 0.0023	9.48E-08
Actinobacteria	0.0009 ± 0.0005	0.0003 ± 0.0002	4.22E-07
Verrucomicrobia	0.0412 ± 0.0315	0.0102 ± 0.0173	2.94E-04
Proteobacteria	0.0208 ± 0.0108	0.0458 ± 0.0270	1.91E-03
Firmicutes	0.3736 ± 0.0614	0.4215 ± 0.0693	6.12E-02
Bacteroidetes	0.5318 ± 0.0691	0.4955 ± 0.0651	NS
Tenericutes	0.0131 ± 0.0075	0.0100 ± 0.0082	NS
Cyanobacteria	0.0006 ± 0.0013	0.0014 ± 0.0024	NS
TM7	0.0047 ± 0.0039	0.0040 ± 0.0021	NS
Other	0 ± 0	0 ± 0	NS

All values are presented as mean ± standard deviation. The phyla are listed in order of significance (Wilcoxon rank sum test).

The most abundant genus was unclassified within the family *S24-7*. While not reaching statistical significance, defeated mice exhibited reduced levels of the taxa compared to controls (*p* = 0.072). More broadly, social defeat altered the composition of 33 of the 92 detected genera (see Supplementary Table S1). Among the most abundant were: *Oscillospira* spp. (control *vs*. defeated: 3.2% *vs*. 5.1%; *p* < 0.001), *Bacteroides* spp. (control *vs*. defeated: 3.1% *vs*. 5.0%; *p* < 0.05), an unclassified genus within the family *Lachnospiraceae* (control *vs*. defeated: 2.1% *vs*. 3.1%; *p* < 0.01), *Akkermansia* spp. (control *vs*. defeated: 4.1% *vs*. 1.0%; *p* < 0.001), *Ruminococcus* spp. (control *vs*. defeated: 2.5% *vs*. 1.8%; *p* < 0.05), *Flexispira* spp. (control *vs*. defeated: 0.3% *vs*. 3.1%, *p* < 0.000), unclassified *Mollicutes* (control *vs*. defeated: 0.2% *vs*. 0.1%, *p* < 0.05), *Paraprevotella* spp. (control *vs*. defeated: 0.6% *vs*. 0.0%; *p* < 0.000), and *Dorea* spp. (control *vs*. defeated: 0.3% *vs*. 0.2%; *p* < 0.005) (Fig. 2c).

To further explore these stressor-induced alterations in the relative abundance of bacterial taxa while considering the multidimensional nature of their relationship, we used a Random Forest algorithm. As such, the algorithm was trained to find predictors for classification (e.g. socially defeated *vs*. control mice) using two thirds of taxonomic data. Misclassification rates, or out-of-bag (OOB) error rate, were calculated using the remaining one third of the data and aggregated from all trees. Random Forest regressions accurately predicted all socially defeated *vs*. control mice for both phyla and genera (overall OOB error rate, 0% for both). The importance or relevance for model construction is provided for each bacterial taxon in Supplementary Tables S2 (phyla) and S3 (genera) (see).

### Abundance of *Akkermansia* and other genera correlate with behavioral metrics of anxiety and depression

**Figure 3:**
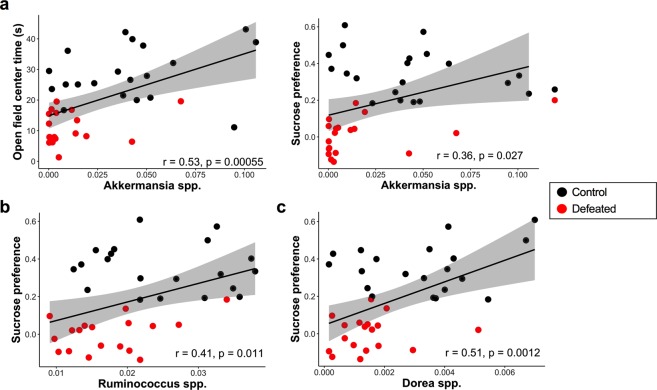
*Akkermansia* spp., *Dorea* spp., and *Ruminococcus* spp. levels correlate with depressive-like behavior. (**a**) Correlation analysis for the relative abundance of *Akkermansia* spp. and time spent in the center of the open field maze (Spearman r = 0.53, *p* < 0.000) (n = 39 pairs) as well as sucrose preference (Spearman r = 0.36, *p* < 0.05) (n = 38 pairs). (**b**,**c)** Analysis also revealed a positive correlation between the relative abundance of both *Ruminococcus* spp. (Spearman r = 0.41, *p* < 0.05) (n = 38 pairs) and *Dorea* spp. (Spearman r = 0.51, *p* < 0.01) (n = 38 pairs) and anhedonia as indexed by sucrose preference.

We assessed whether the alterations in bacterial genera in defeated mice were linked to the presentation of stress-related behavior, namely, anxiety and depression. Analysis returned a positive correlation between the relative abundance of *Akkermansia* spp. and time spent in the center of the open field (Spearman r = 0.53, *p* < 0.001) as well as sucrose preference (Spearman r = 0.36, *p* < 0.05), a measure of depressive-like behavior (Fig. 3a). The abundances of *Oscillospira* spp. (Spearman r = −0.43, *p* < 0.01; Spearman r = −0.43, *p* < 0.01), an unclassified genus within the family *Lachnospiraceae* (Spearman r = −0.33, *p* < 0.05; Spearman r = −0.49, *p* < 0.01), and *Flexispira* spp. (Spearman r = −0.59, *p* < 0.0001; Spearman r = −0.59, *p* < 0.0001) correlated negatively with both open field center time and sucrose preference (see Supplementary Fig. S4). *Bacteroides* spp. correlated negatively with open field center time (Spearman r = −0.4, *p* < 0.05), but not with measures of depressive-like behavior (see Supplementary Fig. S4). Conversely, the abundances of *Ruminococcus* spp. (Spearman r = 0.41, *p* < 0.05) and *Dorea* spp. (Spearman r = 0.51, *p* < 0.01) positively correlated with the severity of anhedonia as assessed with sucrose preference, but not with measures of anxiety-like behavior (Fig. 3b,c).

### Prediction of metagenomic function content

To further investigate the potential functional implications of these structural shifts in gut microbial composition, predictions were made on 16S rRNA-derived OTUs using PICRUSt^17^. A total of 16 KEGG pathways were found to be differentially represented between the control and defeated groups (corrected *p* (FDR) < 0.05; see Supplementary Table S4). The analysis in the defeated group indicated a lower frequency of pathways involved in G protein-coupled receptors (GPCR), steroid biosynthesis, and fluorobenzoate degradation. Defeated mice also exhibited increased abundance of pathways involved in alpha-linolenic acid metabolism, electron transfer carriers, flavone and flavonol biosynthesis, bacterial motility proteins and chemotaxis, Parkinson’s disease, and Prion diseases.

### Exposure to mild chronic social defeat stress produces stress-resilient and stress-susceptible mice that exhibit differences in microbial composition

**Figure 4:**
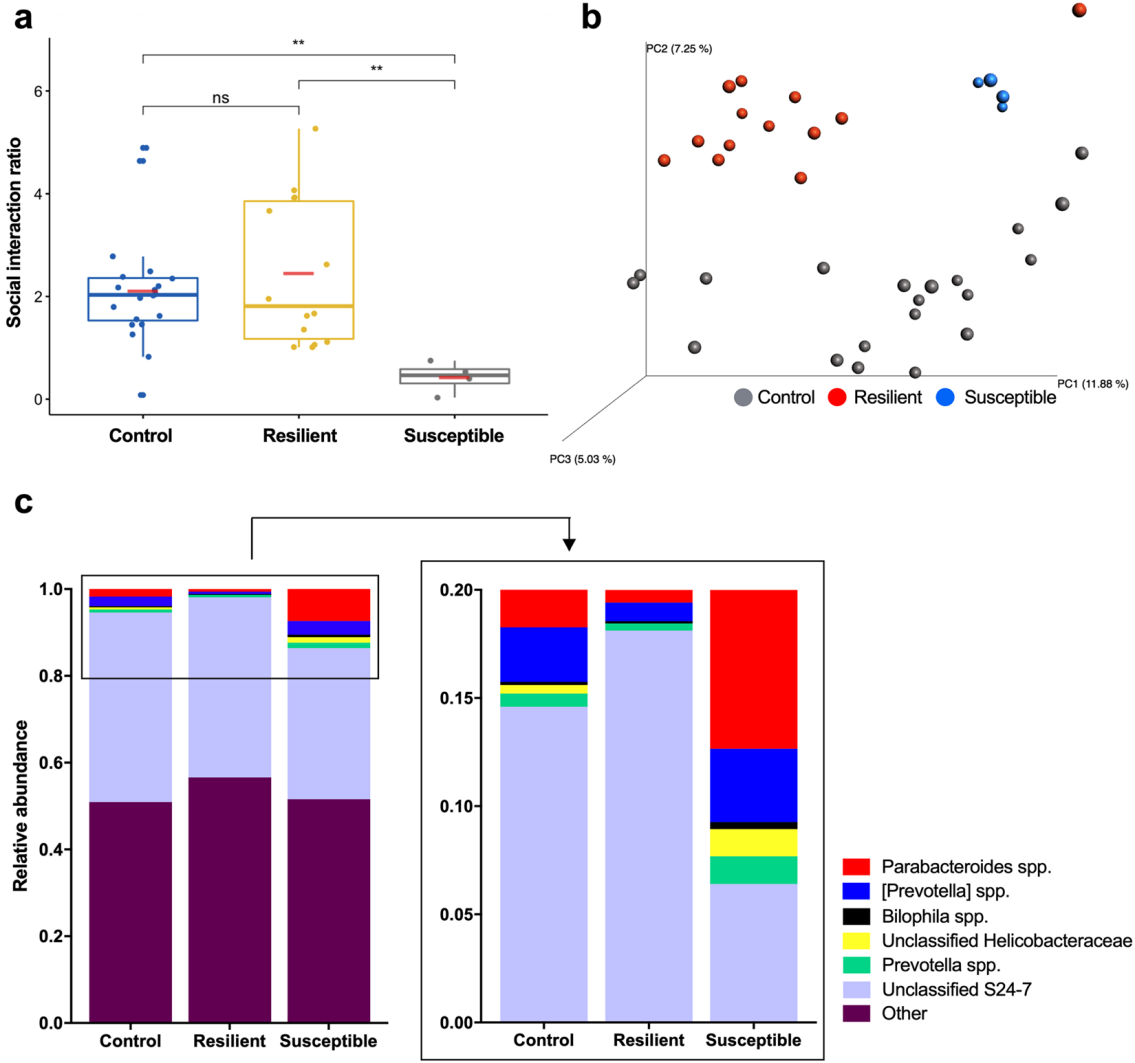
Exposure to chronic mild social defeat stress induces shifts in microbial communities and alters the abundance of relevant taxa. (**a**) Social interaction ratios (time in the interaction zone in the presence of a novel target mouse divided by time in this zone in the absence of a target mouse) of the non-stressed control, resilient, and susceptible mice. (**b**) Principal coordinate analysis of the microbiome community compositions in non-stressed control (n = 19), resilient (n = 14), and susceptible (n = 4) mice. (**c**) Taxonomic distribution at the genus level of fecal samples derived from the control and defeated groups. Susceptible mice displayed increases in *Prevotella* spp. (green) and an unclassified genus within the family *Helicobacteraceae* (yellow) relative to both control and resilient animals. Susceptible mice also revealed significant increases in *Parabacteroides* spp. (red) relative to resilient, but not control, animals.

In addition to examining the global effects of social defeat, we stratified defeated mice into resilient and susceptible subpopulations in accordance with well-established guidelines^16^. Among the 20 mice that experienced social defeat, 14 had social interaction ratios ≥ 1 and were classified as resilient (70%), 4 had social interaction ratios < 1 and were classified as susceptible (20%), and 2 mice—while included in the two-group analysis—were excluded from further resilient/susceptible classification due to aberrant behaviors, such as spending no time in the social or non-social zone. While our milder social stress produced fewer susceptible animals than expected^16^, the social interaction ratios of susceptible mice (0.43 ± 0.15) differed significantly from both the resilient (2.45 ± 0.39) and non-stressed control (2.11 ± 0.25) animals (*p* < 0.001) (Fig. 4a).

Both resilient and susceptible mice exhibited comparable levels of anxiety- and depressive-like behaviors (see Supplementary Fig. S5). Compared with non-stressed controls, both resilient and susceptible mice exhibited reductions in distance traveled (*p* < 0.001 and *p* < 0.05, respectively), while only the resilient animals showed decreased vertical activity (*p* < 0.05) during the open field test. Both resilient and susceptible mice exhibited anxiety-like behavior, as demonstrated by decreased center time (*p* < 0.0001 and *p* < 0.01, respectively) and increased corner time (*p* < 0.0001 and *p* < 0.01). Resilient and susceptible animals also displayed significant increases in depressive-like behaviors, such as anhedonia and despair, as evidenced by decreased sucrose preference (*p* < 0.0001 and *p* < 0.01, respectively). However, only the resilient animals exhibited increased immobility time in the forced swim test (Day 1: *p* < 0.05; Day 2: *p* < 0.05).

We next explored the abundance of the microbial communities among non-stressed control, resilient, and susceptible mice as classified by their social interaction ratios (Table 2). Notably, unweighted PCoA plots revealed distinct clustering of microbiota (999 permutations, non-parametric *p* < 0.05) (Fig. 4b). These results were confirmed using the ANOSIM test on unweighted Unifrac distances (10,000 permutations, *p* < 0.0000). Further examination of the differences between the susceptible and resilient subpopulations revealed alterations in the abundances of 12 bacterial genera (see Supplementary Table S5). Notably, susceptible mice displayed increases in *Prevotella* spp. (control *vs*. resilient *vs*. susceptible: 1.7%, 0.9%, 7.4%; *p* < 0.05) and an unclassified genus within the family *Helicobacteraceae* (control *vs*. resilient *vs*. susceptible: 0.4%, 0.0%, 1.4%; *p* < 0.05) relative to both control and resilient animals (Fig. 4c). Susceptible mice also revealed significant increases in *Parabacteroides* spp. (control *vs*. resilient *vs*. susceptible: 0.6%, 0.3%, 1.4%; *p* < 0.05) relative to resilient, but not control, animals.

**Table 2:** Relative abundances of bacterial phyla in control *vs*. resilient *vs*. susceptible mice exposed to chronic mild social defeat stress.

Phylum	Control (n = 19)	Resilient (n = 14)	Susceptible (n = 4)	*p* value
Deferribacteres	0 ± 0 ^A^	0.0021 ± 0.0021^B^	0.0005 ± 0.0008^B^	9.13E-07
Actinobacteria	0.0009 ± 0.0005 ^A^	0.0003 ± 0.0001^B^	0.0004 ± 0.0002^B^	2.53E-05
Verrucomicrobia	0.0412 ± 0.0315 ^A^	0.0139 ± 0.0200^B^	0.0014 ± 0.0015^B^	2.32E-03
Proteobacteria	0.0208 ± 0.0108 ^A^	0.0410 ± 0.0291^B^	0.0640 ± 0.0172^B^	3.55E-03
Tenericutes	0.0131 ± 0.0075 ^A^	0.0113 ± 0.0086 ^A^	0.0028 ± 0.0020^B^	4.00E-02
Cyanobacteria	0.0006 ± 0.0013	0.0007 ± 0.0011	0.0038 ± 0.0043	NS
Firmicutes	0.3736 ± 0.0614	0.4151 ± 0.0687	0.3895 ± 0.0209	NS
Bacteroidetes	0.5318 ± 0.0691	0.5026 ± 0.0636	0.5224 ± 0.0140	NS
Other	0 ± 0	0 ± 0	0 ± 0	NS
TM7	0.0047 ± 0.0039	0.0038 ± 0.0022	0.0047 ± 0.0012	NS

All values are presented as mean ± standard deviation. The phyla are listed in order of significance (Kruskal-Wallis test). Means not sharing the same superscript are significantly different at p < 0.05.

Further exploration of these stress-resilient and stress-susceptible relationships with Random Forest regression revealed an OOB error rate for predicting control *vs*. resilient *vs*. susceptible mice of 16.22% for phyla and 13.3% for genera with errors largely attributable to the susceptible mice being classified as resilient. The importance for each bacterial taxon is provided in Supplementary Tables S6 (phyla) and S7 (genera).

## Discussion

Here, we found that exposure to a milder, seven-day iteration of chronic social defeat stress resulted in robust shifts in the structural composition of fecal microbiota. These findings were consistent with previous reports by Galley and colleagues that as little as 2 hours of social stress alters the community of mucosa-associated microbes^9^. Moreover, our data suggest that many of these alterations in the relative abundances of bacteria are correlated with the severity of anxiety- and depressive-like behaviors. By contributing to the discussion of which bacterial taxa are most relevant to the interconnectivity between gut microbiota and behavior, these data lay the groundwork for future, causal research aimed at the identification and understanding of potential targets for psychobiotics^18^.

Our results corroborate recent literature describing alterations to the structure of microbial communities following exposure to social stressors^8,11,19^. Rather than a phylum-level binary shift in the ratio of *Firmicutes* to *Bacteroidetes*, a measure often seen as indicative of the ‘health’ of intestinal microbiota, we observed both increases and decreases in the abundance of OTUs within each of the major phyla^20^. The nuance and complexity of microbial community structure were also evident at lower taxonomic ranks. Focusing on the family *Lachnospiraceae*, defeated animals displayed increases in the relative abundance within an unclassified genus as well as decreases in the abundances evident in the genera *Ruminococcus* and *Dorea*. While these genera have only recently been described, a study by Bailey *et al*.^7^ suggested that reductions in *Dorea* spp. following exposure to social stress were inversely related to levels of circulating proinflammatory cytokines, including interleukin-6 and monocyte chemoattractant protein-1, both of which are elevated in patients with depressive symptomatology^21^. Our data support this relationship as the relative abundance of *Dorea* spp. was positively correlated with depressive-like behavior.

Of particular interest, these data align with recent reports of reductions in the relative abundance of *Akkermansia* in socially stressed animals^12,14^. Here, we showed that mice with low levels of fecal *Akkermansia* spp. exhibited decreased center time during the open field test, which is indicative of increased anxiety-like behavior, as well as decreased sucrose preference, suggesting increased anhedonia, compared to animals with higher levels of the bacteria. This pairs with studies demonstrating that administration of a prebiotic increases the relative abundance of *Akkermansia* following exposure to chronic social stress^22,23^. Similar increases in *Akkermansia* were found following administration of minocycline, which has been shown to be an effective, adjunctive treatment for depression^24^. *Akkermansia* has also received attention for its role in gut barrier function and permeability as well as protection from intestinal inflammation^24^. Members of the genus have demonstrated the ability to produce acetate and propionate as products of mucus degradation^25^. Importantly, production of these short-chain fatty acids appears localized within the mucus layer, close to epithelial cells, which serve as the interface for interaction between gut microbiota and host tissues^26,27^. To this end, given its abundance in healthy mucosa, members of the genus *Akkermansia* have been suggested as biomarkers for a healthy intestine. Recent studies have detailed an inverse correlation between the abundance of *Akkermansia* and several intestinal disorders, including inflammatory bowel disease, Crohn’s disease, ulcerative colitis, and appendicitis^28–30^. Critically, as proteome analyses of human fecal samples indicate a high proportion of *Akkermansia muciniphila* mucus-degrading proteins expressed *in vivo*, discussion of *Akkermansia* has relevance for human health and disease states^31^.

Our analyses predicted reduced frequency of signaling molecule pathways, including those involving GPCRs. This finding is substantiated by overwhelming clinical, genetic, and pharmacological evidence that GPCRs play critical roles in the pathophysiology of stress-related and mood disorders^32^. For example, GPCR kinases and β-arrestins, both major regulators of GPCR signaling, have been implicated in both the mechanisms of depression as well as the actions of antidepressant drugs^33^. Abilify, one of the best-selling drugs globally, acts as a partial agonist/antagonist for GPCRs^34^.

Although we observed comparable levels of anxiety and depression between susceptible and resilient mice, and the overall microbial composition was similar between the two, we did observe some microbial differences unique to susceptible mice. In particular, susceptible mice displayed significant increases in *Prevotella* spp. relative to both resilient and control animals as well as significant increases in Parabacteroides spp. relative to resilient, but not control, mice. This finding echoes recent clinical reports detailing alterations in the proportion of *Prevotella* spp. within fecal microbial communities of patients with MDD that were consistent with self-reported depression severity^35^. Similarly, *Parabacteroides* spp. was relatively more abundant in a patient population with active MDD compared to healthy controls^6^. As such, fecal microbiota become valuable characteristic parameters in not only the diagnosis, but also the surveillance, of patients with MDD and other stress-related disorders.

Of note, our efforts were limited to evaluating the microbial community in fecal matter. Recent literature suggests a differential impact of stress on the composition of luminal and mucosal-associated communities^8^. These results emphasize the need for follow-up research to consider the potentially compartmentalized nature of microbial shifts in order to most accurately understand and represent how such alterations might impact health and disease states.

We have also considered other possible sources for the microbial alterations in the defeated animals. While analyzing fecal samples collected during the 24-hour period prior to social defeat and following a period of co-housing, we noticed an emergence of microbial profiles that appeared cage-specific (see Supplementary Fig. S6). Since mice were randomized to different conditions, this cage effect did not extend to our main analyses examining social defeat vs. controls. We did wonder though whether there might be an effect of co-housing socially defeated mice with a CD1. To examine the possibility that the microbiota of CD-1 mice were transferred to C57BL/6J mice during the defeat sessions or the co-housing period that followed, we examined the fecal microbiota of the 30 CD-1 mice used during experimentation. Compared with the fecal OTUs of the C56BL/6J mice, 159 of the 243 OTUs were detected in both mouse groups, and 55 and 29 OTUs were C57BL/6J- and CD-1-specific, respectively (FDR < 0.05). To visualize the potential differences between the mice in terms of the relative abundances of the fecal bacterial composition, the distances between the communities were assessed with an unweighted Unifrac PCoA, and the relative abundances of the bacterial phyla and genera were examined (see Supplementary Fig. S6). The relative abundances of the phyla and genera that appeared differentially abundant among the control and defeated animals were examined with respect to CD-1 microbial profiles (see Supplementary Tables S8 and S9). Moreover, the Random Forest OOB error rate for predicting CD1 *vs*. control *vs*. defeated mice was 1.45% for both phyla and genera, which was due to a single CD1 mouse being misclassified as a defeated C57BL/6J mouse. The importance for each bacterial taxon is provided in Supplementary Tables S10 (phyla) and S11 (genera). Taken, as a whole, marked differences existed between the microbial compositions of the CD-1 and C57BL/6J mice. There were, however, instances in which the relative abundances of the bacterial phyla and genera differed between the C57BL/6J controls and CD-1s but not between the CD-1s and C57BL/6J defeated animals. In this sense, while it appears likely that the alterations observed between the control and defeated animals were an effect specifically resulting from chronic mild social defeat, it is also possible that a small number of these differences may have stemmed from the acquisition of the CD-1 microbiome through either co-housing or coprophagy.

Altogether, these data indicate that the microbiome may play a role in the pathogenesis of anxiety and depression. Here, we identified microbial shifts relevant to stressor exposure and stress-responsivity that expand the examination of stress-related disorders to include the role of the gastrointestinal system and the resident microbiota that are likely co-chaperones in the impact of the environment on brain-based health and disease outcomes. While our data point to an association between shifts in microbial composition resulting from exposure to a mild stressor that in turn correlate with measures of anxiety- and depressive-like behavior, future research is required to determine whether these shifts are causal in nature. Future investigation is also required to tease apart the partially overlapping behavioral phenotypes of stress-resilient and stress-susceptible animals. Of note, while both resilient and susceptible mice displayed increases in anxiety- and depressive-like behaviors relative to controls, there were microbial shifts specific to susceptible animals. As such, perhaps the emergence of microbiota-based differences precedes shifts in behavioral outcomes. Taking this into account, implementation of clustering algorithms, like k-means clustering, could be instrumental in evaluating behavioral differences among groups stratified based on taxonomic differences. Incorporation of this data-driven approach would also more directly lend to identification of particular bacterial species driving differences in stress responsivity as well as *in vitro* and *in vivo* manipulation of these communities.

## Materials and Methods

### Animals

All animals were handled according to the National Institutes of Health Guide for the Careand Use of the Laboratory Animals, and the experiments were conducted according to a protocol approved by the Duke University Animal Care and Use Committee. Male C57BL/6J and CD-1 (6 and 8 weeks old, respectively) were purchased from The Jackson Laboratory (Bar Harbor, ME) and housed in conventional mouse cages (30 × 18 × 12 cm, Allentown, Inc., Allentown, PA). Mice were maintained on a modified reverse light cycle, with a 12-hr/12-hr light-dark cycle (lights on at 1400 h) with food (Prolab® RMH 3500, LabDiet, St. Louis, MO) and water available *ad libitum*. Social defeat stress was started after at least one week of acclimatization. Unless otherwise specified, the behavioral interventions were performed within 1 hr after the onset of the dark cycle, and periods up to 5 hr were allowed for data collection. The CD-1 mice were individually housed using paper huts (Bio-Hut^TM^, Bio-Serv, Flemington, NJ) and enriched with approximately 25 g of shredded Enviro-dri® paper (Shepherd Specialty Papers, Milford, NJ) until the start of experimentation. The C57BL/6J mice were housed five per cage, and the animals were randomly assigned to the control or experimental groups in order to minimize cage effects. Some measurements were not available for all mice participating in this study due to technical issues (e.g., low read count during sequencing and software difficulties during behavioral tasks). N values are specified in the text where relevant.

### Social defeat stress model

In order to facilitate high levels of attack behavior, the CD-1 mice were screened against each other at 0700 h, approximately 1 hr after the onset of the dark cycle (See Figs 1a and S7). To minimize the physical wounding of the defeated mice, a previous chronic social defeat protocol^16^ was adapted such that the procedure lasted seven consecutive days and the defeat sessions were shortened from 10 min to a maximum of 5 min. Briefly, C57BL/6J mice were individually introduced to the home cages of resident CD-1 retired breeders, and interactions were permitted for 5 min or until attacks and bites occurred continuously for 30 s. The defeat sessions were recorded using StereoScan (CleverSys, Inc., Reston, VA). The latency to first attack, frequency of attacks and bites, and whether or not the C57BL/6J mice showed behaviors indicative of counterattack or resistance, such as lunging or tail rattling, were recorded. Following defeat, the mice were separated by a perforated divider that allowed the transmission of visual and olfactory cues but prevented all physical contact between the animals. For this 24-hr period of pair-housing, both mice had access to food and water, but only the CD-1 males had approximately 10 g of their home-cage nesting materials returned. This procedure was repeated for seven consecutive days with the C57BL/6J mice exposed to a new CD-1 retired breeder each day. For the duration of the defeat procedure, the control C57BL/6J mice were pair-housed but separated by a perforated divider to prevent physical contact with cage mates. The control mice were rotated to a new cage with a new control cage mate daily. Twenty-four hr after the last social defeat or control session, all mice were individually housed for the remainder of the study.

### Social interaction test

Twenty-four hr after the conclusion of the defeat sessions, social avoidance behaviors were examined using the social interaction test, as previously described^16^. Testing took place in a dimly lit room using two identical open field arenas (42 cm^3^). A wire-mesh enclosure with a Plexiglas frame (10 × 6 × 42 cm) was positioned against one of the four walls. During the baseline trial, this enclosure was empty. During the social trials, the enclosure housed a CD-1 retired breeder that had been screened for aggressive behavior but had not defeated any of the C57BL/6J mice. All sessions used center-point tracking and were videotaped with the EthoVision 11.5 video-tracking system (Noldus Information Technology bv, Wageningen, The Netherlands). Recording began when the nose and center of the mouse were detected in the arena and ended automatically after 150 s. Social interaction ratios were calculated as the time spent in the interaction zone (14 × 24 cm) in the presence of the CD-1 retired breeder divided by the time in this zone in the absence of the CD-1 breeder. Mice with social interaction ratios ≥ 1 were classified as resilient to the effects of the defeat stressor, whereas mice with ratios < 1 were classified as susceptible, as previously described^16,36^.

### Open field test

Open field testing was performed based on methods previously reported for mice^37^. Briefly; the mice were tested individually in VersaMax Legacy open field arenas (21 × 21 × 30 cm, Omnitech Electronics, Inc., Columbus, OH). Activity was monitored by two rows of 16 photobombs that were located around the perimeter of the open field and spaced 2.5 cm apart. The arenas were connected to a computer running Fusion activity software (Omnitech Electronics, Inc.), which was used to monitor distance travelled (cm) and vertical activity. In addition, the activity in various areas of the arena, such as the center, perimeter, and corners, was monitored. The mice were allowed free exploration for 1 hr. Between tests, the arenas were cleaned with LabSan 256Q (Sanitation Strategies, Holt, MI) and dried.

### Forced swim test

Forced swim testing was conducted based on methods described by Fukui *et al*.^38^. Testing began when the animal was placed in a glass beaker (4 L volume, 15 cm diameter) filled with water maintained at room temperature (22 °C) for 6 min. All sessions were recorded to digital video (30 frames/s) using a camera (Panasonic CCTV WV-BP330, Panasonic Corporation, Osaka, Japan) that was interfaced to a computer with MediaRecorder software (Noldus Information Technology bv). Videos were later scored for immobility using EthoVision 11.5. Mobility states were scored continuously and averaged over 10 frames/s with thresholds of immobility set at 8%. Testing took place over two consecutive days.

### Sucrose preference test

The preference of the mice for 2% sucrose solution compared to water was examined using methods based on Fukui *et al*.^38^. The mice were individually housed in standard cages. To monitor water consumption, two polycarbonate water bottles (Animal Care Systems Inc., Centennial, CO) filled with approximately 300 mL of water were weighed and then hung inside the cage. The bottle weights were recorded daily at approximately the same time. The mice were allowed to drink from the two water bottles for 2–3 days to ensure that no strong bias was present for either bottle. As testing began, a 2% sucrose solution (Superfine Quick Dissolve 100% Pure Cane Sugar, Domino Foods, Inc., Yonkers, NY) was added to one of the two bottles. The mice were allowed to drink freely for 24 hr before the bottles were weighed and then returned to the cage with their positions counterbalanced for an additional 24 hr. Sucrose preference was determined using a discrimination ratio that was calculated by subtracting the total water consumed from the total sucrose consumed and then dividing by the total fluid consumed. Positive scores indicated a preference for sucrose over water, while negative scores indicated a preference for water over sucrose. Scores approaching zero indicated no preference.

### Fecal bacterial analysis

Twenty-four hr before the start of the social defeat trials and 24 hr after the social interaction testing, fecal pellets (1–2 mL) were collected. The samples were promptly frozen and stored at −80 °C until processing. DNA was isolated from 75 mg of fecal pellets according to a fecal sample-based adaptation to a Maxwell® RSC PureFood GMO and Authentication Kit (Catalog No. AS1600; Promega Corporation, Madison, WI), as previously described^39^. Concentrations were determined fluorometrically using the QuantiFluor® ONE dsDNA System (Promega Corporation) on a Quantus^TM^ fluorometer (Promega Corporation), and purity was assessed via 260/280 and 260/230 absorbance ratios as determined by spectrophotometry (Epoch^TM^, BioTek Instruments, Inc., Winooski, VT). After processing, the samples were stored at −20 °C until sequencing.

The V3 and V4 hypervariable regions of the 16S ribosomal RNA (rRNA) gene were amplified from extracted DNA using primer pair sequences (Integrated DNA Technologies, Inc., Coralville, IA) flanked by Illumina overhang adapter sequences (Forward overhang: 5′TCGTCGGCAGCGTCAGATGTGTATAAGAG-ACAG, Reverse overhang: 5′GTCTCGTGGGCTCGGAGATGTGTATAAGAGACA-G). Following polymerase chain reactions (PCRs) to amplify the 16S locus and purification of the PCR products with AMPure XP beads (Beckman Coulter, Inc., Indianapolis, IN), Illumina® sequencing adapters (Illumina, Inc., San Diego, CA) and dual-index barcodes were added to a 50-μL PCR reaction from 5 μL of amplicon PCR product for eight cycles using 2X KAPA HiFi HotStart ReadyMix (Kapa Biosystems, Inc., Wilmington, MA). The amplicons were purified, pooled in equimolar concentrations, and mixed with 20% PhiX control library (Illumina, Inc.). Sequencing was performed on a MiSeq (Illumina, Inc.) using the MiSeq v3 reagent kit (Illumina, Inc.).

### Sequencing data analysis

Sequencing outputs from the Illumina MiSeq platform were converted to the fastq format and demultiplexed using Illumina bcl2fastq (v2.18.0.12, Illumina, Inc.). The resulting paired-end reads were joined using the QIIME 1.9.0^40^ invocation of fastq-join^41^ with default parameters. The index and linker primer sequences were trimmed, and the reads were subsequently filtered for quality by removing any reads in which less than 70% of the quality scores fell below the quality score threshold of 24. The quality control of both the raw and processed sequencing reads was verified by FastQC (v0.11.2, Babraham Institute, Cambridge, UK). The sequences were clustered into OTUs based on the *de novo* OTU picking algorithm using the QIIME implementation of UCLUST^42^ at a similarity threshold of 97%. OTUs identified as chimeric by vsearch^43^ of the ChimeraSlayer ‘gold’ reference database^44^ and those composed of a single read (singletons) were eliminated. The remaining OTUs were assigned taxonomic identifiers according to the Greengenes database^45^, their sequences were aligned using template alignment through PyNAST^46^, and a phylogenetic tree was built with FastTree 2.1.3^47^. Alpha diversity, with respect to Chao1, observed species, and the Shannon index, was estimated using QIIME at a rarefaction depth of 10,000 sequences per subsample. Beta diversity estimates were calculated within QIIME using the weighted and unweighted Unifrac distances^48,49^ between the samples at a subsampling depth of 10,000. As such, one sample from a control mouse, which failed to reach 10,000 reads, was excluded from analysis of bacterial relative abundance.

### Random Forest regressions

To determine the extent to which the different levels of bacteria optimally classified mice, we ran Random Forest regressions using the Feather Spray randomForest package (v. 4.6–14) in R (version 3.5.1, 2018–07–02; R Foundation for Statistical Computing, Vienna, Austria). The total number of trees was set at 500, and the minimum number of variables tested at each split was set to the square root of the number of predictors included for each bacterial level. OOB estimates of the error rate for each predictor model were described as well as the relative importance for each predictor.

### Statistical analysis

The analyses were performed using R (version 1.0.143) software. Quantitative variables were expressed as mean and standard deviation. Variables were compared using non-parametric tests, including the Wilcoxon Rank Sum and Kruskal-Wallis tests followed by a Dunn’s post-test. For all analyses, the threshold for significance was two-tailed, and *p* values ≤ 0.05 were considered statistically significant.

### Accession codes

are available through the Sequencing Read Archive such that all deep sequencing data upon which the manuscript is based can be accessed in full: SRP152351.

## Supplementary information

Supplementary Figures

Supplementary Tables
