# Supplementary material for: Relative abundance of Akkermansia spp. and other bacterial phylotypes correlates with anxiety- and depressive-like behavior following social defeat in mice
Source: Sci Rep. 2019 Mar 1;9:3281. doi: 10.1038/s41598-019-40140-5 (PMC6397238; doi:10.1038/s41598-019-40140-5)
Supplement: Supplementary file 1 — Supplementary Figures [file 41598_2019_40140_MOESM1_ESM.docx]

**Relative abundance of *Akkermansia* spp*.* and other bacterial phylotypes correlates with anxiety- and depressive-like behavior following social defeat in mice**

Kara D. McGaughey^1,*^, Tulay Yilmaz-Swenson^1^, Nourhan M. Elsayed^1^, Dianne A. Cruz^1^, Ramona M.. Rodriguiz^1,2^, Michael D. Kritzer^1^, Angel V. Peterchev^1,3,4,5^, Jeffrey Roach^6^, William C. Wetsel^1,2^, Douglas E. Williamson^1,7^

^1^Department of Psychiatry and Behavioral Sciences, Duke University Medical Center, Durham, NC, 27710, USA

^2^Mouse Behavioral and Neuroendocrine Analysis Core Facility, Duke University Medical Center, Durham, NC, 27710, USA

^3^Department of Biomedical Engineering, Duke University, Durham, NC, 27708, USA

^4^Department of Electrical and Computer Engineering, Duke University, Durham, NC, 27708, USA

^5^Department of Neurosurgery, Duke University School of Medicine, Durham, NC, 27710, USA

^6^Research Computing, University of North Carolina at Chapel Hill, Chapel Hill, NC, 27599, USA

^7^Durham VA Medical Center, Durham, NC, 27705, USA

*kara.mcgaughey@pennmedicine.upenn.edu


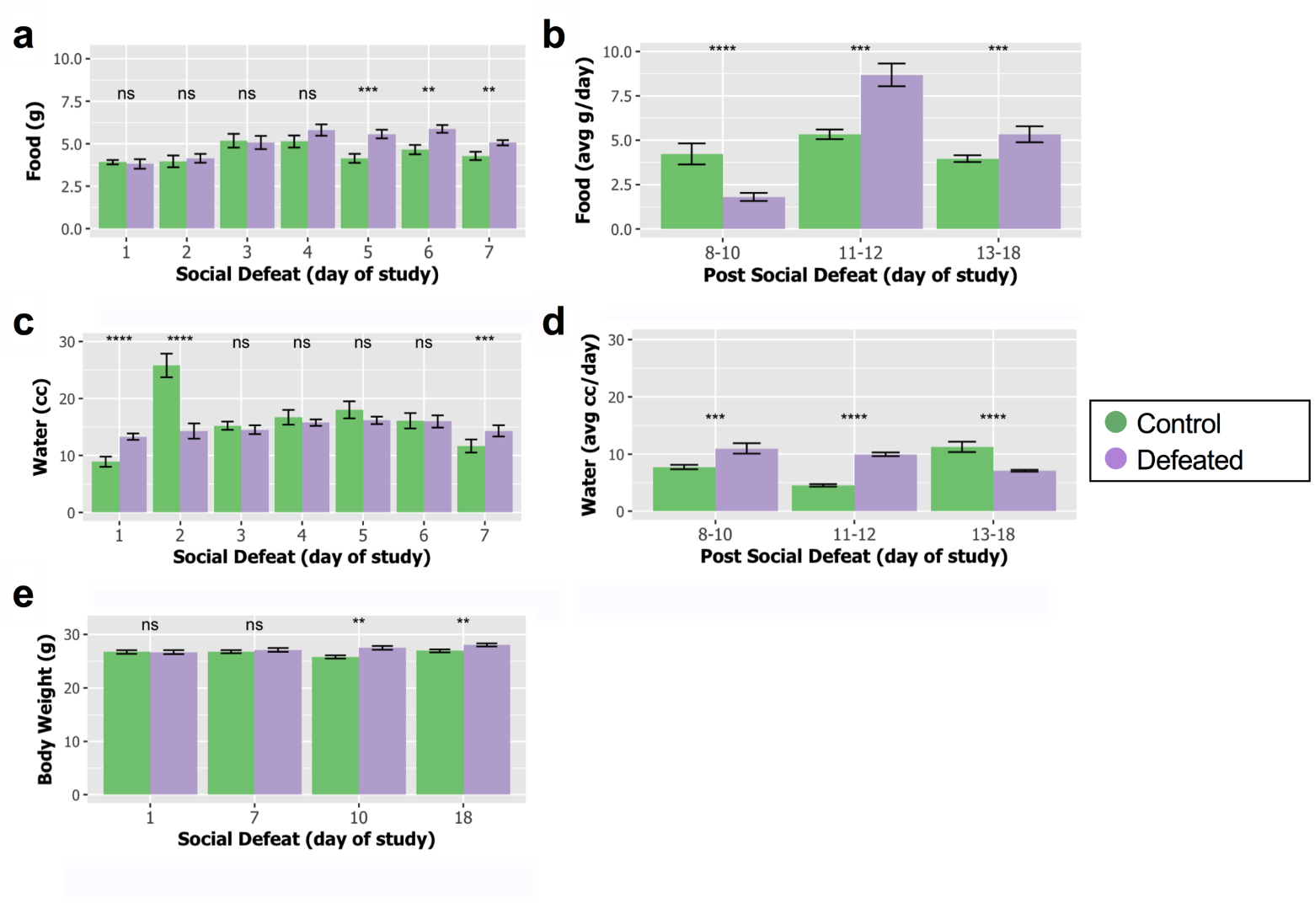


**Supplementary Figure S1. Variability in food and water intake was observed across exposure to chronic mild social defeat stress and behavioral testing.** (**a**-**d**) There was a significant interaction between condition and day of social defeat for both food (F_6,266_ = 2.26, *p* < 0.04) and water (F_6,264_ = 9.83, *p* < 0.0001). Similarly, during the behavioral testing phase, there was a significant interaction between condition and day of social defeat for both food (F_6,112_ = 22.62, *p* < 0.0001) and water (F_6,113_ = 39.19, *p* < 0.0001). Significant pairwise comparisons between control and defeated mice for each day are depicted. (**e**) The control and defeated mice had similar weights pre- and post-social defeat, but the defeated mice weighed significantly more during the behavioral testing phase.

All pairwise comparisons were made using the Mann-Whitney U test: ns, not significant; *, *p* < 0.05; **, *p* < 0.01; ***, *p* < 0.001; and ****, *p* < 0.0001.

**
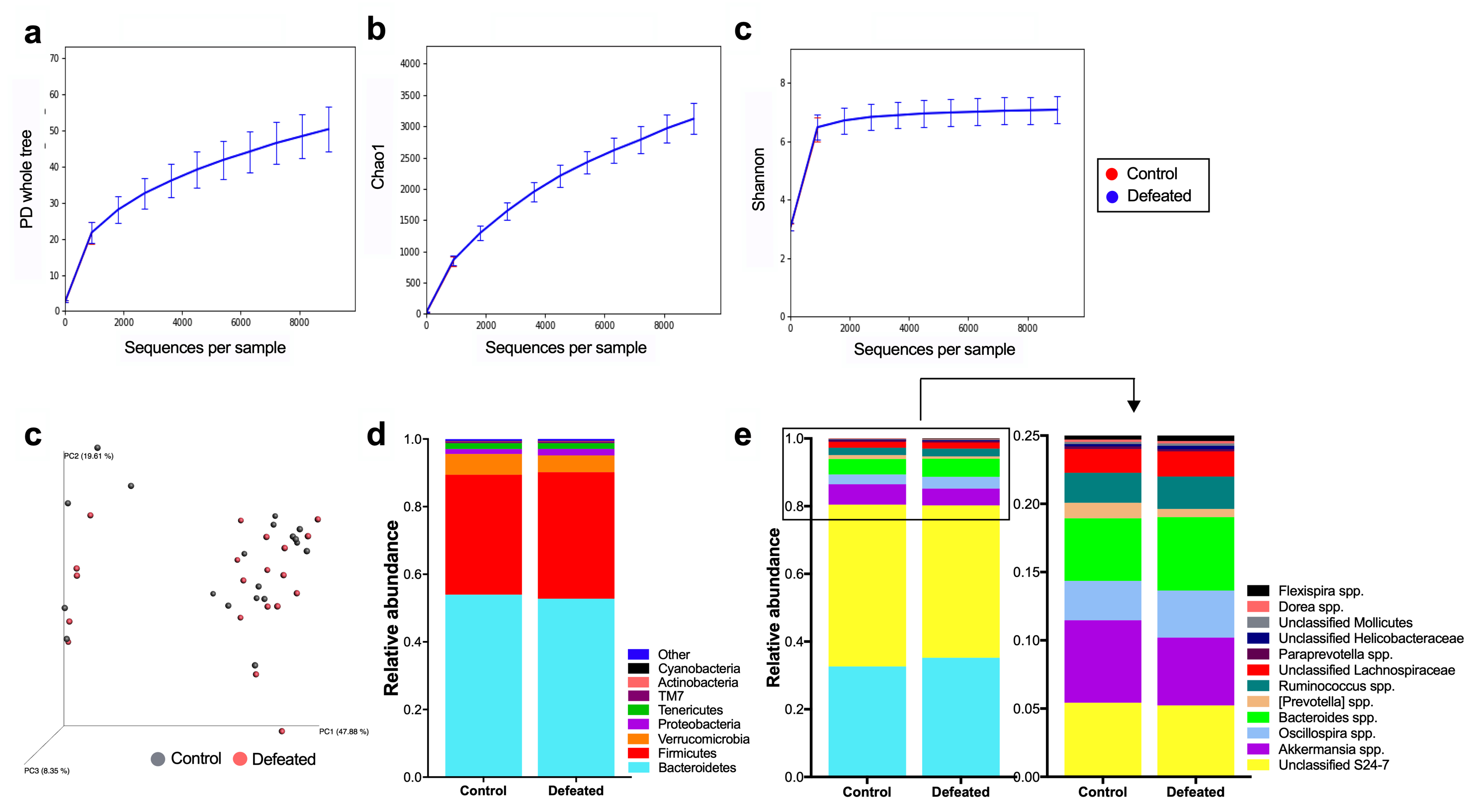
**

**Supplementary Figure S2. Fecal sample analyses show similar structural compositions of the microbiota of the to-be control and to-be defeated mice at baseline.** (**a**) Alpha diversity metrics of to-be control (n = 20) and to-be defeated animals (n = 20) at baseline revealed no differences in the phylogenetic diversities [phylogenetic diversity (PD) whole tree, *p* = 0.938], Chao1 richness estimates (*p* = 0.676), or characterization of species diversities (Shannon index, *p* = 0.832). (**b**) Principal coordinate analysis of both weighted (non-parametric *p* = 0.449) and unweighted (non-parametric *p* = 0.560) Unifrac distances confirmed the similarities of the community structures between the to-be control (n = 20) and to-be defeated (n = 20) C57BL/6J mice. A weighted Unifrac principal coordinate analysis is shown here. (**c**) Representative graph of the relative abundances of the bacterial communities at the phylum taxonomic rank among the to-be control and to-be defeated mice. The Wilcoxon rank sum analysis revealed that none of the eight detected phyla differed significantly between the groups. (**d**) Representative graphs of the relative abundances of the bacterial communities at the genus level among the to-be control and to-be defeated mice. The Wilcoxon rank sum analysis revealed that none of the 74 detected genera differed significantly between the groups.


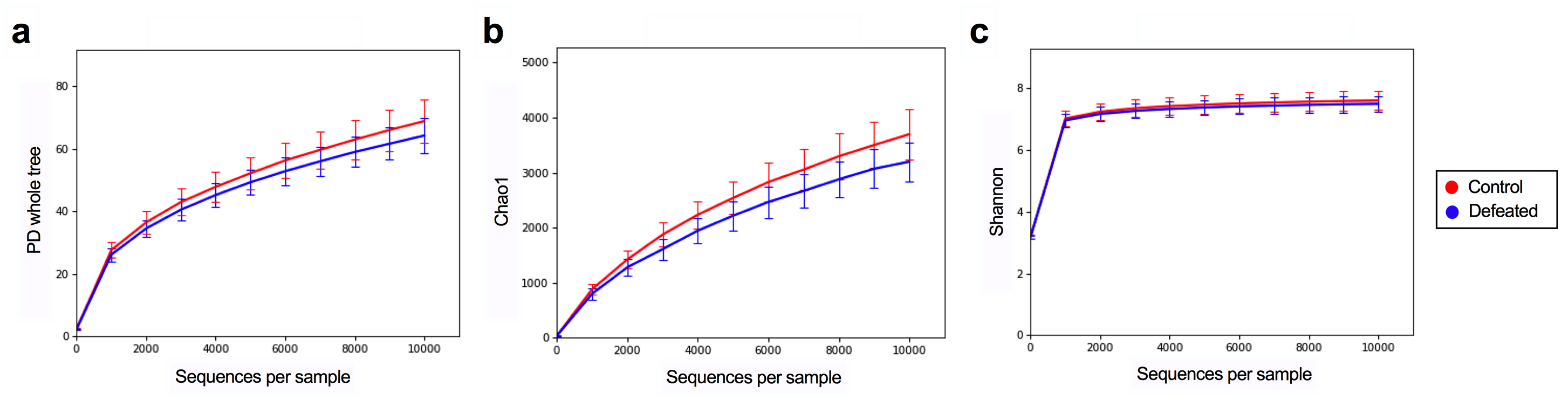


**Supplementary Figure S3. Fecal sample analyses show that exposure to chronic mild social defeat alters the structural compositions of the microbiota.** (**a**) Phylogenetic diversity (PD) whole trees of the control (n = 20) and defeated (n = 19) groups (*p* < 0.05). (**b**) Chao1 richness estimates for the microbial communities of the control (n = 20) and defeated (n = 19) groups (*p* < 0.005). (**c**) Characterization of the species diversities of the microbial communities of the control (n = 20) and defeated (n = 19) groups with the Shannon index (*p* = 0.29).


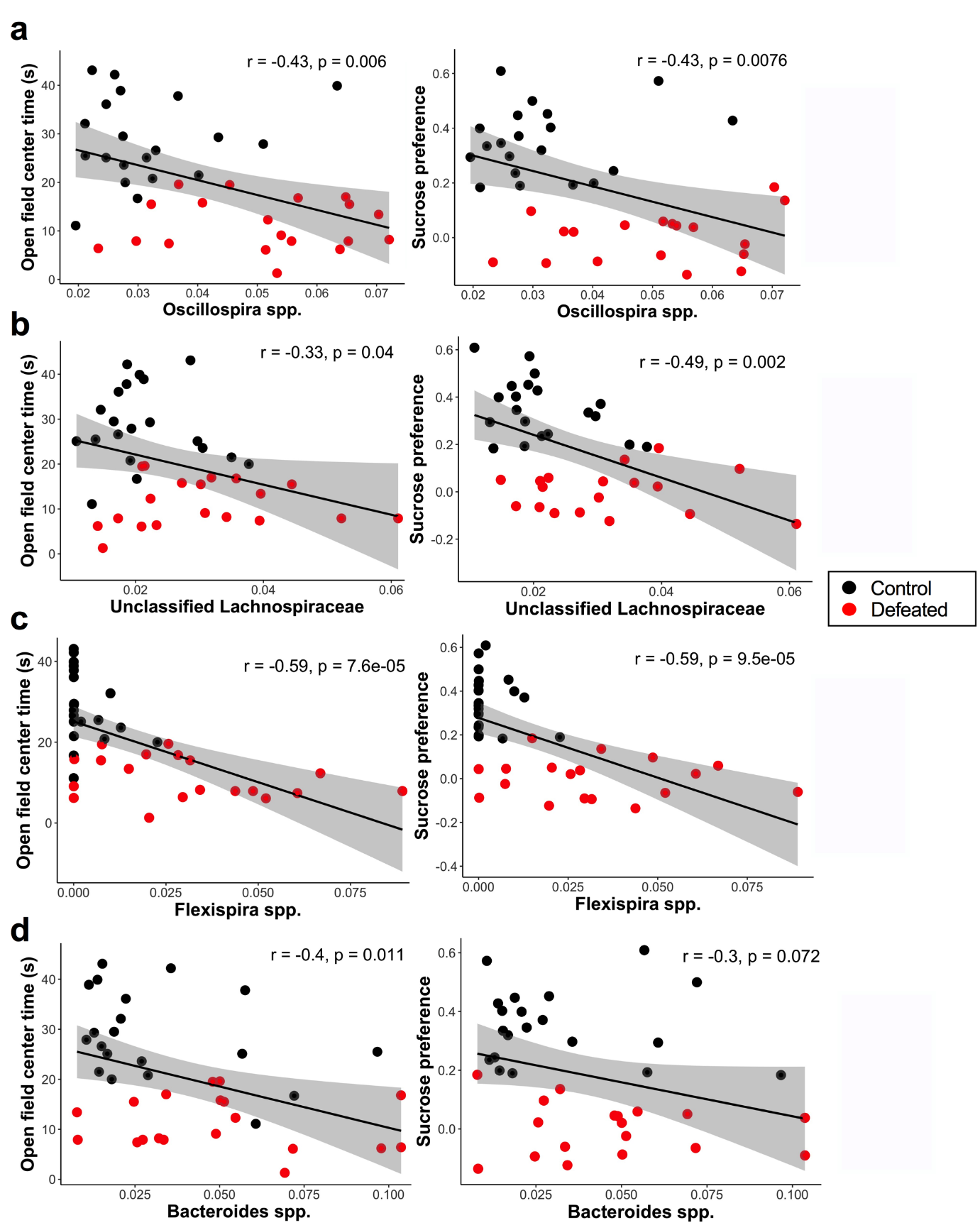


**Supplementary Figure S4. Abundances of several bacterial genera correlate with anxiety- and depressive-like behavior.** (**a**) Correlation analysis for the relative abundance of *Oscillospira spp.* and time spent in the center of the open field (Spearman r = -0.43, *p* < 0.01) as well as sucrose preference (Spearman r = -0.43, *p* < 0.01). (**b**) Correlation for the relative abundance of an unclassified genus within the family *Lachnospiraceae* and open field center time and sucrose preference (Spearman r = -0.33, *p* < 0.05; Spearman r = -0.49, *p* < 0.01). (**c**) Correlation for the relative abundance of *Flexispira spp.* for time spent in the open field center and sucrose preference (Spearman r = -0.59, *p* < 0.0001; Spearman r = -0.59, *p* < 0.0001). (**d**) Correlation for the relative abundance of *Bacteroides spp.* and open field center time (Spearman r = -0.40, *p* < 0.05) as well as sucrose preference (Spearman r = -0.30, *p* = 0.072).


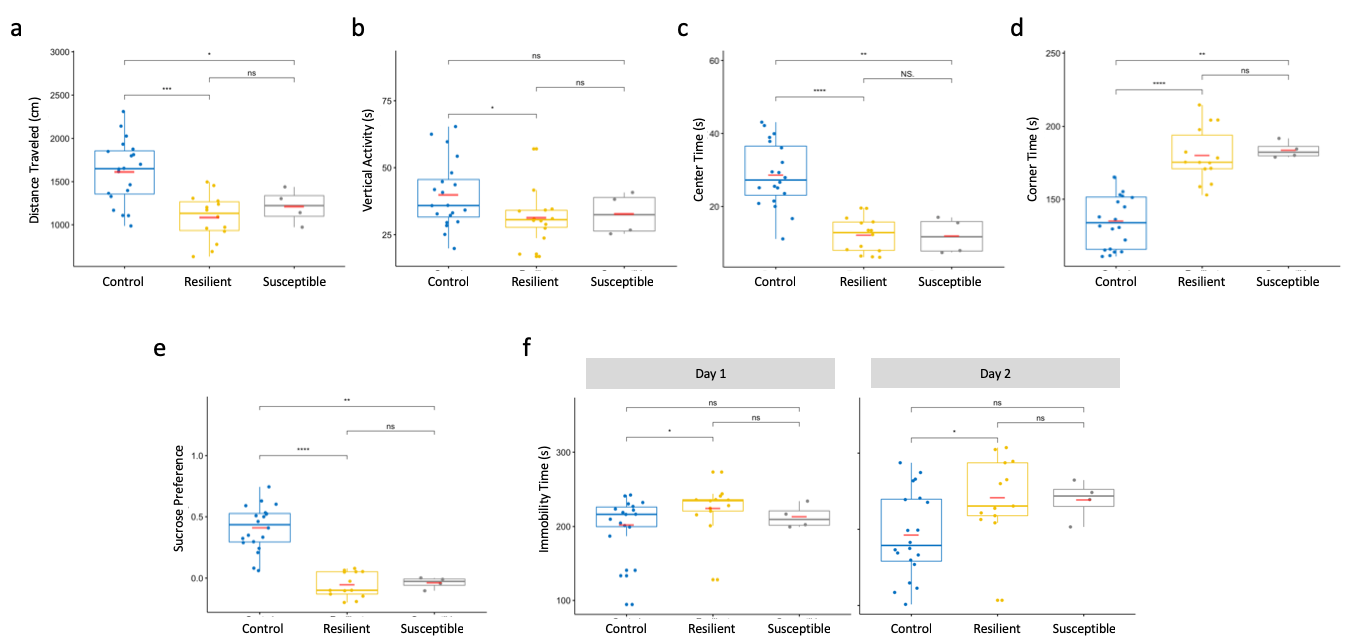


**Supplementary Figure S5. Both resilient and susceptible mice exhibit anxiety- and depressive-like behavior.** (**a-d)** Quantification of behavior during the open field test. (**e**) Sucrose preference. (f) Immobility behavior during the two-day forced swim test. For all graphs: n = 20 control, 14 resilient, and 4 susceptible; Wilcoxon rank sum test: *, *p* < 0.05; **, *p* < 0.01; ***, *p* < 0.001; ****, *p* < 0.001; ns, not significant. The lines in the center of the boxes indicate median values, while the lower and upper box boundaries indicate 1^st^ and 3^rd^ quartiles, respectively. The red lines indicate mean values.


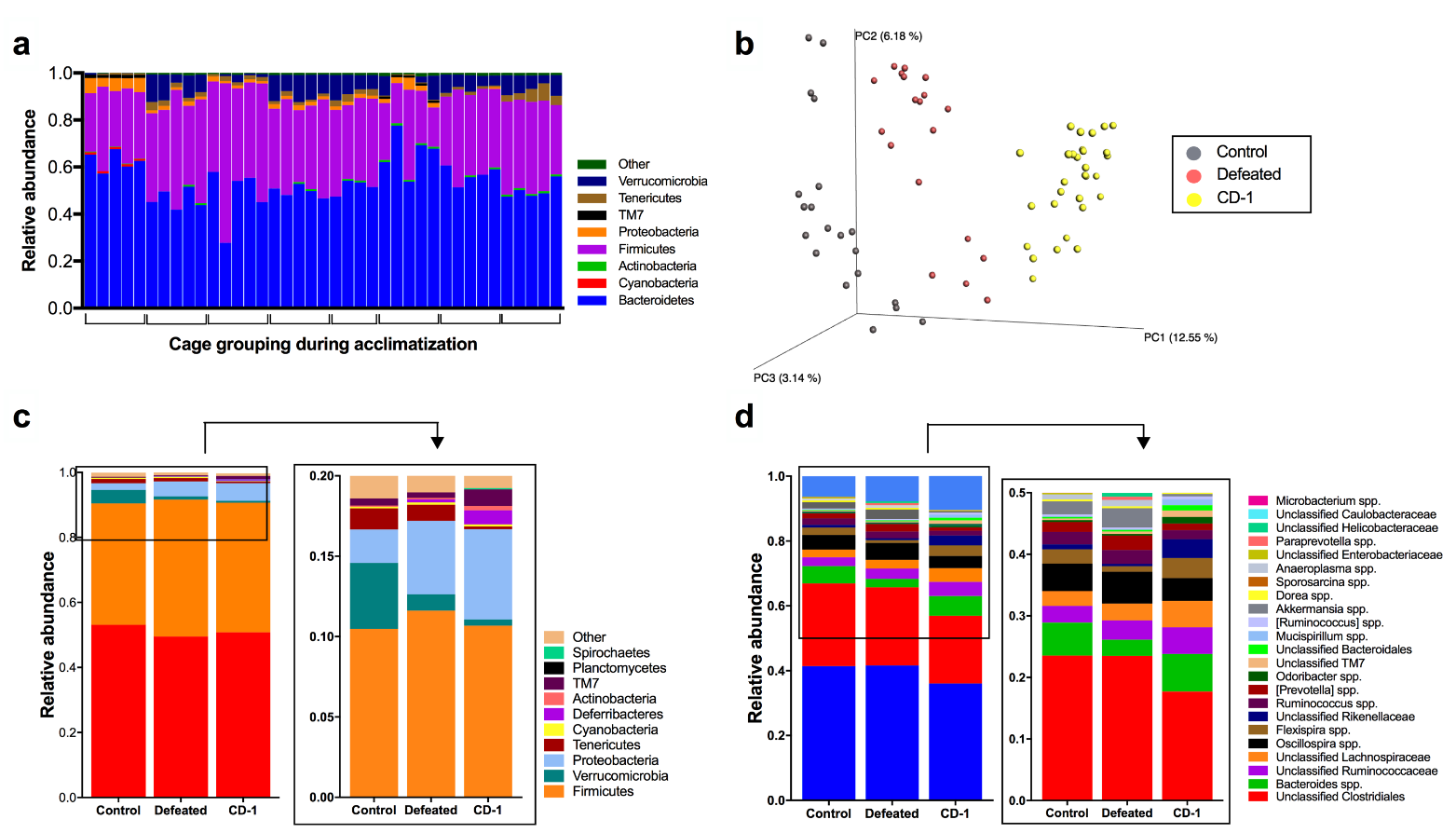


**Supplementary Figure S6. Fecal sample analyses show different microbial profiles of Defeated and CD-1 mice.** (**a**) Bacterial phylum distribution in individual subjects (n = 39); representative of the microbial composition as assessed 24 hr before the start of social defeat. During acclimatization to the animal vivarium, the mice were housed in groups of five as shown on the x-axis. The emergence of a microbial profile unique to each cage is visible. (**b**) Principal coordinate analysis of the microbiome communities in the non-stressed control C57BL/6J mice (gray) (n = 19), defeated C57BL/6J mice (red) (n = 20), and aggressor CD-1 mice (yellow) (n = 30). (**c**) Representative graphs of the relative abundances of the bacterial communities at the phylum taxonomic rank among the control, defeated, and CD-1 mice. (**d**) Representative graphs of the relative abundances of the bacterial communities at the genus level among the control, defeated, and CD-1 mice.


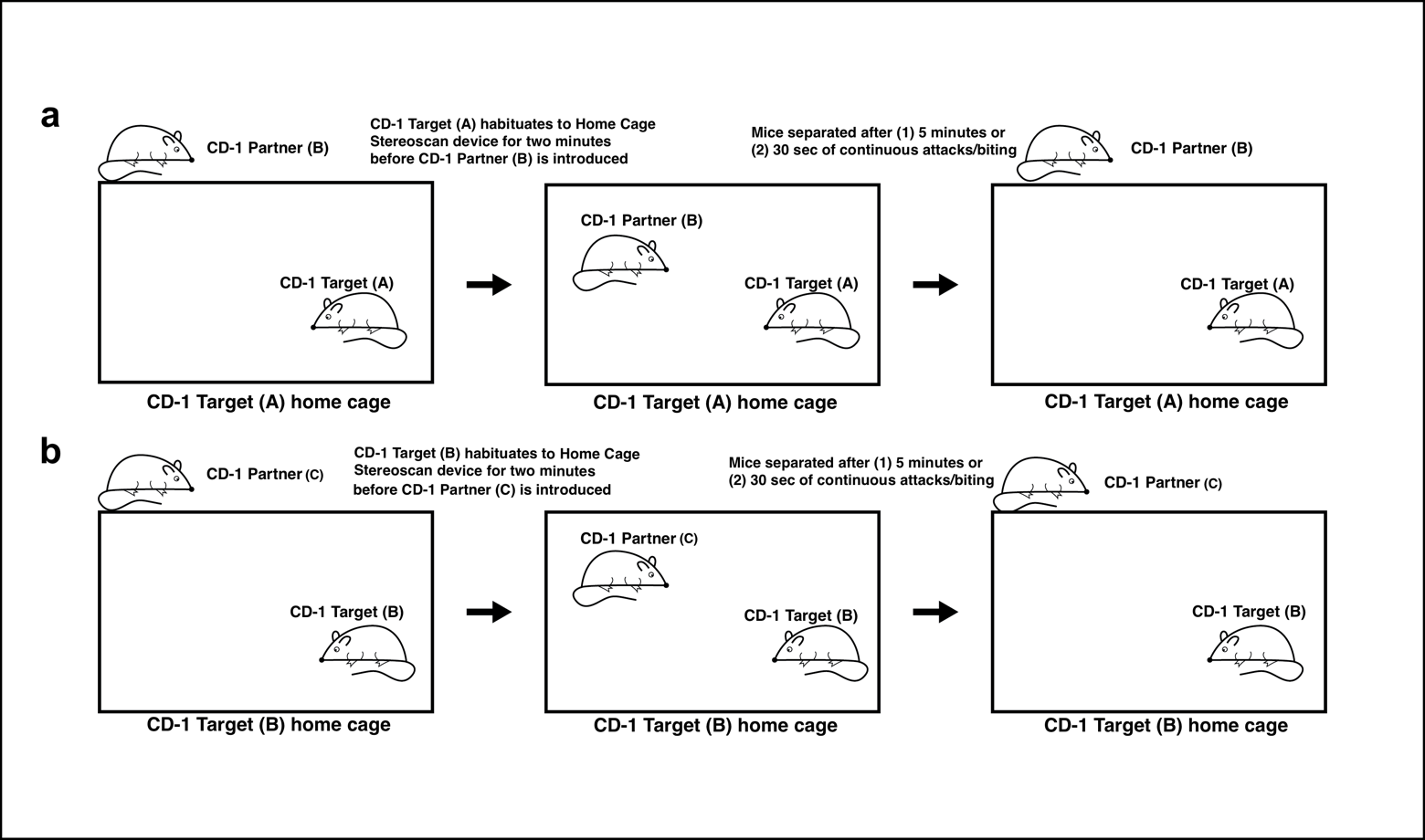


**Supplementary Figure S7. Experimental design of the social defeat stress model.** (**a**) For a screening session, the home cage of a CD-1 male designated as a “Target” mouse was placed into a CleverSys Home Cage StereoScan device (CleverSys, Inc., Reston, VA). After a 2-min habituation, a second CD-1 (the “Partner”) was introduced. The mice were allowed to interact for up to 5 min or until 30 sec of continuous attacks or biting behavior was observed. (**b**) The following day, mice that served as Partners became Targets, and pairs were assigned such that the mice had not previously interacted. This two-day process was repeated for a total of four consecutive screening days in order to facilitate high levels of attack behavior.
